# Hormone receptors expression in ovarian cancer taking into account menopausal status: a retrospective study in Chinese population

**DOI:** 10.18632/oncotarget.20251

**Published:** 2017-08-14

**Authors:** Fang Shen, Xuyin Zhang, Yiqun Zhang, Jingxin Ding, Qi Chen

**Affiliations:** ^1^ The Hospital of Obstetrics & Gynaecology, Fudan University, Shanghai, China; ^2^ Department of Obstetrics & Gynaecology, The University of Auckland, Auckland, New Zealand

**Keywords:** ovarian cancer, subtypes, ER positivity, PR positivity, menopause

## Abstract

Ovarian cancer is a major gynaecological cancer with different subtypes and studies have suggested that estrogen receptor (ER) or progesterone receptor (PR) positivity are associated with better clinical outcomes. Furthermore, the clinical outcomes of ovarian cancer are better in Asian compared to Caucasian. To date, studies investigating the ER or PR positivity in all subtypes of ovarian cancer, including borderline epithelial, are limited. In this retrospective study we investigated ER and PR positivity in Chinese women with malignant epithelial ovarian cancer (n=577), sex cord-stromal tumor (n=26) and borderline epithelial ovarian cancer (n=98) taking into account menopausal status. The positivity of ER (>85%) or PR (>58%) was higher in serous and endometrioid carcinoma of malignant epithelial ovarian cancer than that in mucinous and clear-cell carcinoma (<19% of ER or 24% of PR). The majority of serous carcinomas of borderline epithelial ovarian cancerwere ER or PR positive, but in contrast less than 33% of mucinous carcinomas of borderline epithelial ovarian cancerswere ERor PR positive.Furthermore, there was no association between the ER or PR positivity and menopausal status in both malignant and borderline epithelial ovarian cancer. We also found that the age at diagnosis with ovarian cancer was younger in Chinese women. Our data suggest that ER or PR positivity in Chinese women with ovarian cancer is similar to that of other ethnicities reported in literature, suggesting that the better clinical outcomes seen in Asian may be associated with other factors such as age at diagnosis of ovarian cancer.

## INTRODUCTION

Ovarian cancer is a major gynaecological cancer with more than 220,000 newly diagnosed cases every year in developed countries in 2014, and is the 5th leading cause of death in women [1]. The exact causes of ovarian cancer are still unclear, however a number of risk factors for developing ovarian cancer such as early menarche and late menopause, nulliparity, obesity, age at menopause, hormone replacement treatment during menopause, and ethnicity have been identified [2–7]. Most of these risk factors are associated with the changes in levels of sex hormones during women's lifetime. Of these sex hormones, estrogen has an effect on ovarian cell proliferation, which is shown by studies suggesting that women who have postmenopausal hormone replacement therapy (HRT) with estrogen for 10 years or longer have an increased risk of developing ovarian cancer [8].

Ovarian cancers are traditionally classified into three subtypes: epithelial ovarian carcinoma, sex cord stromal tumors and germ cell tumors based on presumed histogenesis and direction of differentiation. Each of these three classifications also includes a series of subtypes [9]. The estrogen receptor (ER) and progesterone receptor (PR) mediate the effects of sex hormones on proliferation and apoptosis of ovarian cancer cells [10]. The association of hormone receptors expression, such as ER or PR, and better survival is well-documented in breast [11, 12] and endometrial cancer [13, 14]. A number of studies have reported that the ER or PR positivity is associated with the prognosis or the treatment of ovarian cancer [15–21]. Although the results from those studies were controversial due to the small sample size and different ethnicities, a recent largest study with 12 international centers reported that the expression of ER or PR is associated with improved survival of subtypes of epithelial ovarian cancer, mainly endometrioid carcinoma and high grade serous carcinoma [22].

The incidence of ovarian cancer and survival including rate and time varies with ethnicity. African American women and Asian women generally have a lower risk of developing ovarian cancer and better clinical outcomes than Caucasian women do [23, 24], but the underlying reasons for this are unclear. Whether the positivity of ER or PR in ovarian cancer in Asian (including Chinese) women who have better clinical outcomes is different with Caucasian women has not been investigated.

In previous studies, the ER or PR positivity has mostly been investigated in epithelial ovarian cancer. Studies investigating the expression of ER or PR in sex cord-stromal tumor and borderline epithelial ovarian cancer are limited. Menopause at an older age is one of the risk factors for developing ovarian cancer. Ovarian cancer is most commonly diagnosed after menopause and the typical age of diagnosis is 63 years (www.cancer.org). Therefore, the current study aimed to investigate the positivity of ER or PR in epithelial ovarian cancer and sex cord-stromal tumors and borderline epithelial ovarian cancer taking into account menopausal status in Chinese population.

## RESULTS

### Clinical characteristics of the study population

The clinical and histological characteristics of study participants with primary malignant ovarian cancer (epithelial ovarian cancer and sex cord-stromal tumor) and borderline epithelial ovarian cancer are summarised in Table 1. The median age of patients at diagnosis with malignant ovarian cancer was 53 (range 16-79) years old. The median age at diagnosis of women with epithelial ovarian cancer was 53 (range 25 to 79) years. The median age at diagnosis of women with sex cord stromal tumors was 45 (rang 16 to 70) years, which was significantly younger than women with epithelial ovarian cancer (p=0.002). The median age at diagnosis in women with malignant ovarian cancer was significantly higher than that in borderline epithelial ovarian cancer (53 ranging from 16 to 79 years versus 36 ranging from 18 to 80 years, p=0.001).

**Table 1:** Clinical characteristics of the study population

	Women with malignant ovarian cancer (N=603)
Age at diagnosis (years, median/range)	53 (16-79)
Epithelial ovarian cancer (number, %)	577 (95.6%)
Serous carcinoma	394 (65.3%)
Low-grade	46 (12%)
High-grade	348 (88%)
Mucinous carcinoma	37 (6.2%)
Endometrioid carcinoma	40 (6.6%)
Clear-cell carcinoma	106(17.5%)
Sex cord-stromal tumor (number, %)	26 (4.4%)
Granulosa cell tumor (number, %)	19 (73%)
Leydig cell tumor (number, %)	7 (27%)
	**Women with borderline epithelial ovarian cancer (N=98)**
Age at diagnosis (years, median/range)	36 (18-80)
Histological type (number, %)	
Serous carcinoma	59 (60.2%)
Mucinous carcinoma	39 (39.8%)

Of 701 patients with primary ovarian cancer, 577 women were diagnosed with malignant epithelial ovarian cancer and 26 were diagnosed with sex cord-stromal tumors, and 98 patients were diagnosed with borderline epithelial ovarian cancer. Of 577 women who were diagnosed with malignant epithelial ovarian cancer, 255 (44%) women were diagnosed before menopause. Of 98 women who were diagnosed with borderline epithelial ovarian cancer, 74 (76%) women were diagnosed before menopause.

### The positivity of estrogen receptor (ER) and progesterone receptor (PR) is different between types of ovarian cancer and subtypes of epithelial ovarian cancer

**Figure 1:**
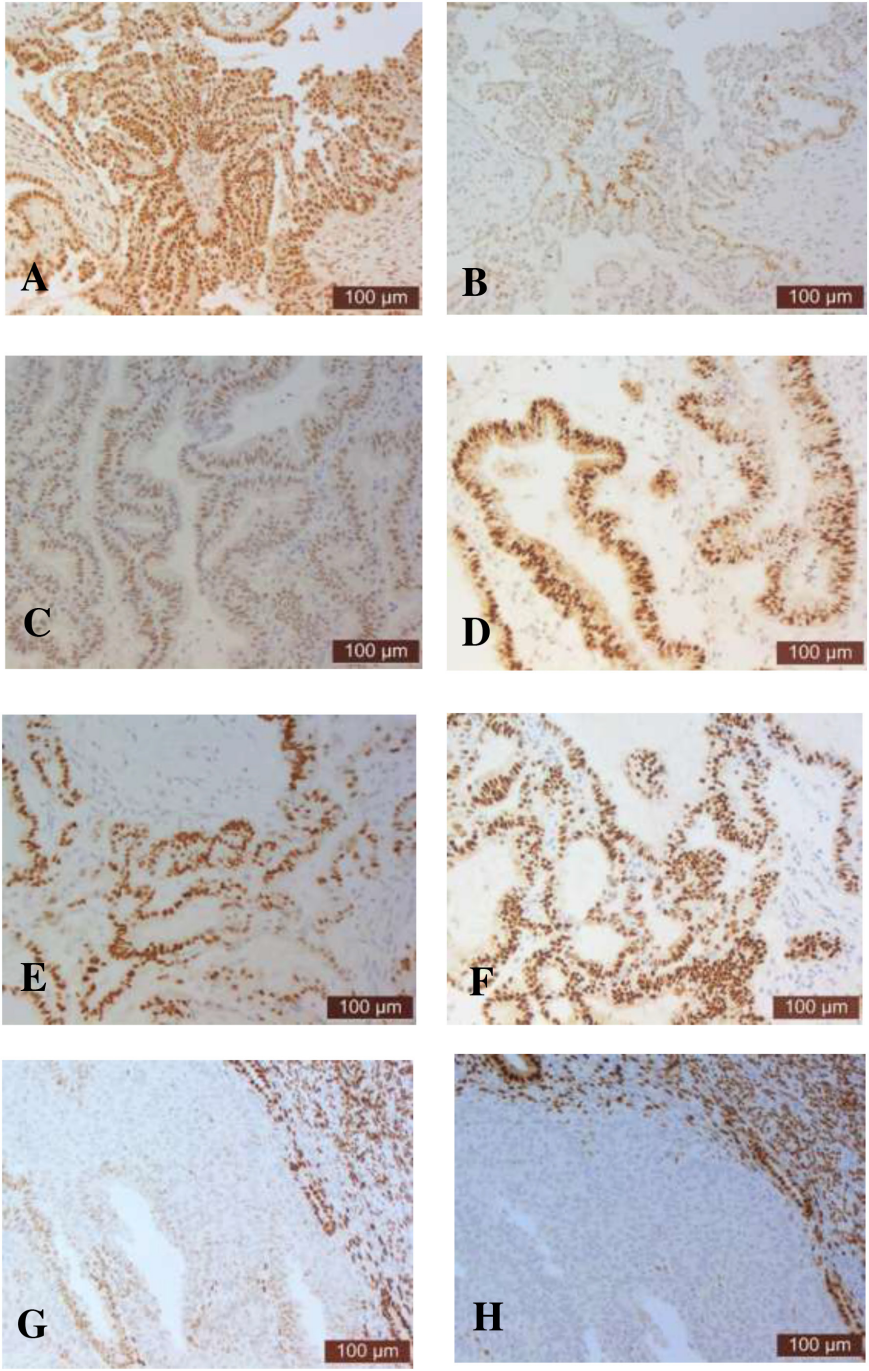
Representative immunohistochemistry images showing the immunostaining of ER and PR in serous **(A-B)**, mucinous **(C-D)**, endometrioid **(E-F)** or clear cell **(G-H)** carcinoma of ovarian cancer.

The expression of ER or PR in serous carcinoma, mucinous carcinoma, endometrioid carcinoma and clear cell carcinoma of ovarian cancer is shown in Figure 1. ER or PR was mainly expressed in the cytoplasm and nucleus. ER and PR positivity differed substantially between the types of ovarian cancer and subtypes of each type of ovarian cancer (Table 2). Overall, the positivity of ER or PR in epithelial ovarian cancer was 69% or 48%, respectively. The positivity of ER or PR in sex cord-stromal tumors was 46% or 80% respectively. There was more ER positive epithelial ovarian cancer, while higher PR positive was seen in sex cord-stromal tumor.

**Table 2:** The number of patients with expression of estrogen receptor (ER) or progesterone receptor (PR) in malignant ovarian cancer according to the subtypes

Epithelial ovarian cancer (n=577)	ER positive (number, %, lower, upper CL)	PR positive (number, %, lower, upper CL)
Serous carcinoma (n=394)	340 (86%) (82.5%, 89.5%)	231(58%) (53.6%, 63.5%)
Mucinous carcinoma (n=37)	7 (18.9%) (7.9%, 35.1%)	9 (24%) (11.7%, 41.2%)
Endometrioid carcinoma (n=40)	34 (85%)(70.1%, 94.3%)	33 (82%) (67.2%, 92.6%)
Clear-cell carcinoma (n=106)	15 (13%) (7.4%, 21.1%)	4 (3.7%) (1.0%, 9.3%)
**Sex cord-stromal tumor (n=26)**	12 (46%) (26.6%, 66.6%)	21 (80%) (60.6%, 93.5%)

CL: Confidence limits.

We then analysed the positivity of ER or PR in subtypes of epithelial ovarian cancer (Table 2). The positivity of ER in serous carcinoma or endometrioid carcinoma was 86% or 85%, respectively, which was higher compared to that of mucinous carcinoma and clear-cell carcinoma (19%, or 13% respectively). The positivity of PR in serous carcinoma or endometrioid carcinoma was 58% or 82%, respectively which was also higher compared to that of mucinous carcinoma and clear-cell carcinoma (24%, or 3.7% respectively).

We further divided serous carcinoma by low-grade and high-grade. The positivity of ER or PR in low-grade of serous carcinoma was 84% (39 out of 46 cases) or 84% (39 out of 46 cases) respectively. The positivity of ER or PR in high-grade serous carcinoma was 86% (300 out of 348 cases) or 55% (191 out of 348 cases) respectively. There was no difference in the positivity of ER between low-grade and high-grade serous carcinomas. However, the positivity of PR in low-grade serous carcinomas was significantly higher than in high-grade serous carcinomas (p=0.001).

### The positivity of estrogen receptor (ER) or progesterone receptor (PR) is not associated with menopausal status in epithelial ovarian cancer

As menopausal status is one of the risk factors for developing ovarian cancer, we then compared the positivity of ER or PR in patients with epithelial ovarian cancer before menopause or after menopause according to the cancer type (Table 3). There was no difference in the frequency of ER or PR positivity in any of the four subtypes of epithelial ovarian cancer between premenopausal and postmenopausal women, except that the frequncy of PR positivity in serous carcinomas was significantly higher in premenopausal women than in post menopausal women (Table 3, p<0.0001).

**Table 3:** The number of patients with expression of estrogen receptor (ER) or progesterone receptor (PR) in epithelial ovarian cancer between cancer types according to menopausal status

Serous carcinoma	Premenopause (n=148)	Postmenopause (n=237)	P value
ER positive (number, %)	131 (88 %)	201 (85%)	0.384
PR positive (number,%)	110 (74%)	117 (49%)	<0.0001
**Mucinous carcinoma**	**Premenopause (n=27)**	**Postmenopause (n=8)**	
ER positive (number)	5 (19%)	2 (40%)	0.647
PR positive (number,%)	6 (22%)	2 (40%)	0.999
**Endometrioid carcinoma**	**Premenopause (n=14)**	**Postmenopause (n=24)**	
ER positive (number,%)	11 (78%)	22 (91%)	0.271
PR positive (number, %)	11(78%)	21 (87%)	0.502
**Clear-cell carcinoma**	**Premenopause (n=36)**	**Postmenopause (n=66)**	
ER positive (number, %)	4 (11%)	10 (15%)	0.591
PR positive (number, %)	2 (18%)	2 (3%)	0.519

9 women from serous carcinoma,2 women from mucinous carcinoma, 2 women from endometrioid carcinoma and 4 women from clear-cell carcinoma had hysterectomy resulting in no information about their menopausal status.

### The positivity of estrogen receptor (ER) and progesterone receptor (PR) did not differ in subtypes of borderline epithelial ovarian cancer

Borderline epithelial ovarian cancer is another subset of epithelial ovarian cancer with more favorable clinical outcomes. Therefore, we then analysed the positivity of ER and PR in borderline epithelial ovarian cancer according to main cancer subtypes (Table 4). The positivity of ER or PR in serous carcinoma was 93% for both receptor types, whereas ER or PR positivity in mucinous carcinomas was 28% or 33% respectively. We also analysed the ER or PR positivity in borderline epithelial ovarian cancer according to menopausal status (Table 5). There was no difference in the frequency of ER or PR positivity in borderline epithelial ovarian cancers (both serous carcinoma and mucinous carcinoma) betwen premenopausal and postmenopausal women.

**Table 4:** The number of patients with expression of estrogen receptor (ER) or progesterone receptor (PR) in borderline epithelial ovarian cancer between cancer types

Borderline epithelial ovarian cancer (n=98)	ER positive (number, %, lower, upper CL)	PR positive (number, %, lower, upper CL)
Serous carcinoma (n=59)	55 (93%) (83.5%, 98.2%)	55 (93%) (83.5%, 98.2%)
Mucinous carcinoma (n=39)	11 (28%) (15%, 44.8%)	13 (33%) (19.0%, 50.2%)

CL: Confidence limits.

**Table 5:** The number of patients with expression of estrogen receptor (ER) or progesterone receptor (PR) in borderline epithelial ovarian cancer between cancer types according to menopausal status

Serous carcinoma	Premenopause (n=47)	Postmenopause (n=10)	P value
ER positive (number, %)	45 (96%)	10 (100%)	0.999
PR positive (number, %)	45 (96%)	10 (100%)	0.999
**Mucinous carcinoma**	**Premenopause (n=27)**	**Postmenopause (n=12)**	
ER positive (number, %)	7 (26%)	4 (33%)	0.763
PR positive (number, %)	8 (30%)	5 (42%)	0.562

2 women from seroun carcinoma of borderline ovarian cancer had hysterectomy resulting in no information about their menopausal status.

## DISCUSSION

Tumor expression of the estrogen receptor (ER) or progesterone receptor (PR) has been shown to be positively associated with the prognosis of gynaecolgical cancers including endometrial cancer [13, 14] and breast cancer [11, 12]. However, the data is less conclusive in ovarian cancer because of inconsistent results from previous studies [18, 25–27]. However, recently a large study involving 12 international centres reported that ER or PR positivity is correlated with improved survial of endometrioid carcinoma and high grade serous carcinoma due to the higher frequency of ER or PR positivity in endometrioid and serous carcinoma [22]. Interestingly, Asian women generally have a lower risk of developing ovarian cancer and better clinical outcomes than Caucasian women do [23, 24]. Whether this is a result of differences in the ER or PR positivity in ovarian cancers from Asain (including Chinese) women has not been investigated. In our current study with a large sample size of Chinese women from a single hospital (largest womens’ hospital in China), a higher frequencies of ER (>85%) or PR (> 58%) positivity were noted in serous and endometrioid carcinoma, whilst mucinous and clear cell carcinoma showed lower frequencies of ER (13-19%) or PR (4-24%) positivity. Women with clear cell carcinoma have a poorer clinical prognosis compared with serous carcinoma [28] which could be due to the lower frequency of ER or PR positivity. Our results are similar with other studies in different ethnicities [22].

To date most studies have focused on investigating the association of ER or PR positivity and prognosis of epithelial ovarian cancer. Sex cord stromal tumors are rare tumors comprising less than 5% of all ovarian malignancies. Although it normally presents at younger age with better clinical outcomes, it depends on age at diagnosis and early stage of cancer [29]. Studies investigating the positivity of ER or PR in sex cord-stromal tumors are limited to a single study focused on granulasa cell tumors [30]. In this study, we found that the majority of sex cord-stromal tumors (including granulosa cell tumors and Leyding cell tumors) were PR positive (80%), whilst 46% of tumours were ER positive. Studies have suggested that PR positivity alone was an important bioligical parameter in relation to cancer prognosis and survival [25, 31, 32]. In endometrial cancer, higher PR positivity has a better prognosis than lower PR positivity [31]. Thus, the higher positivity of PR in sex cord-stromal tumors could be also one of the reasons that these tumours have better clinical outcomes.

Although borderline epithelial ovarian cancer (with its two major histologic tumor subtypes, serous carcinoma and mucinous carcinoma) is another subset of epithelial ovarian cancer that has better clinical outcomes, the frequency of ER or PR positivity has not previously been well studied. One previous study showed 75% of cases were ER positive, and 67% of cases were PR positive [20], however that study did not take into account the further subtypes of borderline epithelial ovarian cancer. In our current study, we found that the majority of serous carcinomas of borderline epithelial ovarian cancer were ER and PR positive (93%), whilst 28% or 33% of women with mucinous carcinoma of borderline epithelial ovarian cancer were ER or PR positive. Interestingly, we also found that PR positivity in both subtypes of borderline epithelial ovarian cancer was higher than that in malignant epithelial ovarian cancer which was consistent with previous findings [20].

Menopause at an older age is one of the risk factors for ovarian cancer. A study suggested that an early age at onset of menopause is negatively correlated with developing ovarian cancer [33]. Studies regarding the association of ER or PR positivity with menopause are limited. In this study, we found that there was no difference in the frequency of ER or PR positivity in any of the four subtypes of epithelial ovarian cancer between premenopausal and postmenopausal women. These findings are consistent with those of a previous study with a smaller sample size [32], as we have confirmed that the frequency of ER or PR positivity is not associated with menopausal status regardless the subtypes of ovarian cancer.

Globally, the majority of women diagnosed with ovarian cancer are diagnosed after menopause between the ages of 60 and 64 years, with 63 years being the average age at diagnosis in developed countries (American Cancer Society 2014). However, the incidence of gynaecological cancers depends on the ethnicity and geographical area [34, 35]. We have recently reported that 44% of Chinese women with endometrial cancer were diagnosed before menopause [36]. In our current study, we interestingly found that 38% women with epithelial ovarian cancer and 76% of women with borderline epithelial ovarian cancer were diagnosed before menopause, suggesting the age at diagnosis with ovarian cancer in Chinese (Asain) women was younger than that reported for Caucasians in the literature [24]. Because we found that the postivity of ER or PR in Chinese women with ovarian cancer is similar to that of other ethnicities, it is important to investigate in the future whether the younger age at diagnosis in Chinese women could explain their better clinical outcomes.

There are some limitations in this study. First, the age of menopause was self-reported by the patients. Secondly, despite the collection of 701 patient samples over a 4 year period in a single women's hospital, the number of endometrioid carcinomas and mucinous carcinomas were still small. To increase the power, the conclusions drawn from this study would need to be further studied with a larger sample size. We also acknowledge that we do not have data available for germ cell tumors, another subtype of ovarian cancer. Third, data on disease progression and survival were not available in this study, however a recent large study involving 12 international centers reported that ER or PR positivity is correlated with improved patient survival in ovarian cancer.

In conclusion, this study with a large sample size has found that the majority of serous carcinomas in both malignant and borderline epithelial ovarian cancers, as well as endometrioid carcinoma in malignant epithelial ovarian cancer, were ER and PR positive in this Chinese population, and the proportion of ER or PR positive tumours were similar to that previously reported in other ethnicities. The ER or PR positivity was not associated with menopausal status. In addition, we found that the age at diagnosis with ovarian cancer was younger in Chinese women compared to that of Caucasians previously reported in literature. Our data suggests that the better clinical outcomes seen in Asian women with ovarian cancer may be associated with other factors such as early age at diagnosis.

## MATERIALS AND METHODS

This study was approved by the Ethics Committee of The Hospital of Obstetrics & Gynaecology, Fudan University of China. All patient-derived tissues were obtained with written informed consent. All methods were performed in accordance with the relevant guidelines and regulations.

### Study participants

In total, there were 847 women with a primary diagnosis of malignant ovarian cancer (epithelial ovarian cancer or sex cord-stromal tumor) and 120 women with a primary diagnosis of borderline epithelial ovarian cancer from January 2012 to December 2015 from The Hospital of Obstetrics & Gynaecology, Fudan University, China. The Hospital of Obstetrics & Gynaecology serves a diverse urban and rural population in China. In this study, retrospective data on 603 women with a primary diagnosis of malignant ovarian cancer and 98 women with a primary diagnosis of borderline epithelial ovarian cancer were available during the 4 year study period. All data including age at diagnosis, self-reported age at menopause, parity and pathological findings of ovarian cancer were collected from the electronic based medical records of patients from the hospital.

Ovarian cancer was classified to serous carcinoma, mucinous carcinoma, endometrioid carcinoma or clear-cell carcinoma according to the classification of the International Federation of Gynaecology and Obstetrics (FIGO). The clinical and histological characteristics of malignant ovarian cancer patients (n=701) including epithelial ovarian cancer with four main subtypes (n=577), sex cord-stromal tumor (n=26), and borderline epithelial ovarian cancer (n=98) are summarized in Table 1.

Ovarian cancer was diagnosed first by a physical examination and followed by other relevant tests such as computed tomography (CT) and magnetic resonance imaging (MRI) scans. The ovarian tissue was examined histologically for characteristics of cancer including subtypes of cancer following the FIGO guidelines.

### Immunohistochemistry

The expression of estrogen receptor (ER) and progesterone receptor (PR) in ovarian tissue (n=701) was measured by immunohistochemistry on paraffin-embedded sections. Briefly, antigen retrieval was performed by treatment with citric acid (pH 6.0) for 20 minutes. Non-specific antibody binding was blocked by incubating with 10% fetal calf serum for 20 minutes. Mouse anti-human ER (1:200) or PR monoclonal antibody (1:000, Dako, Shanghai, China) were added for 1 hour at room temperature. Sections were then washed with phosphate-buffered saline (PBS, PH7.2) and incubated with biotinylated anti-mouse IgG (Dako, Shanghai, China) for 30 minutes. After washing, sections were then incubated with streptavidin-conjugated horseradish peroxidase (Dako, Shanghai, China) for 30 minutes. The antigen–antibody complexes were visualised using 3,3-Diaminobenzidine (DAB) and counterstained with haematoxylin. ER or PR positive cells were serially counted across the tumor tissue. Ovarian cancer cells with a nuclear reaction were considered as positive. The numbers of positive and negative cancer cells were counted and ER or PR positivity was represented as percentage of positive cell. A cut-off point of 1% was used to consider a tumor as ER or PR positive.

### Statistical analysis

The number of patients with positive estrogen or progesterone receptor expression was presented as percentage with lower and upper confidence limits (CL), which express the lower end of the interval and upper end of the interval. The statistical difference in the number of patients with positive estrogen or progesterone receptor expression with subtypes of ovarian cancer in premenopausal and postmenopausal women was assessed by the Chi-square test or Fisher's exact using the Prism software package (GraphPad Software Inc, San Diego, CA, USA) with *p* <0.05 being considered as statistically significant.
